# Changes in subcellular localization reveal interactions between human cytomegalovirus terminase subunits

**DOI:** 10.1186/1743-422X-9-315

**Published:** 2012-12-21

**Authors:** Jian Ben Wang, Yali Zhu, Michael A McVoy, Deborah S Parris

**Affiliations:** 1 Department of Pediatrics, Virginia Commonwealth University, P.O. Box 163, MCV Station, Richmond, VA, 23298-0163, USA; 2 Department of Molecular Virology, Immunology, and Medical Genetics, Ohio State University, Columbus, OH, 43210, USA

**Keywords:** Herpesvirus, Cytomegalovirus, Terminase, DNA packaging, Genome maturation

## Abstract

**Background:**

During herpesvirus replication, terminase packages viral DNA into capsids. The subunits of herpes simplex virus terminase, UL15, UL28, and UL33, assemble in the cytoplasm prior to nuclear import of the complex.

**Methods:**

To detect similar interactions between human cytomegalovirus terminase subunits, the orthologous proteins UL89, UL56, and UL51 were expressed in HEK-293 T cells (via transfection) or insect cells (via baculovirus infection) and subcellular localizations were detected by cellular fractionation and confocal microscopy.

**Results:**

In both cell types, UL56 and UL89 expressed alone were exclusively cytoplasmic, whereas UL51 was ~50% nuclear. Both UL89 and UL56 became ~50% nuclear when expressed together, as did UL56 when expressed with UL51. Nuclear localization of each protein was greatest when all three proteins were co-expressed.

**Conclusions:**

These results support inclusion of UL51 as an HCMV terminase subunit and suggest that nuclear import of human cytomegalovirus terminase may involve nuclear import signals that form cooperatively upon subunit associations.

## Background

Herpesviruses have large (130 - 235 kb) linear double-stranded DNA genomes that replicate via concatemeric intermediates consisting of head-to-tail linked genomes. An enzyme complex called terminase functions to package the DNA into preformed capsids and cleave the concatemers at precise locations to release unit length genomes within the capsids [1]. The herpes simplex virus type 1 (HSV-1) proteins UL15, UL28, and UL33 are believed to comprise the HSV-1 terminase. Studies have also detected interactions between UL56 and UL89 [2,3], the human cytomegalovirus (HCMV) orthologs of HSV-1 UL28 and UL15, respectively. The possible inclusion of UL51 as part of the HCMV terminase has been inferred as it is an ortholog of HSV-1 UL33. Here, we show evidence that co-expression of UL56 with UL89 or UL56 with UL51 is necessary for nuclear import of UL56 or UL89 in the absence of any other viral proteins. These results suggest that UL51 is a component of the HCMV terminase and that specific interactions among terminase subunits in the cytoplasm are prerequisites for its nuclear importation.

## Results

Each subunit was expressed using recombinant baculoviruses. PCR overlap extension was used to modify the 5’ end of the UL51 ORF to encode an N-terminal FLAG epitope. Sf9 insect cells were infected with 3 pfu/cell of each baculovirus individually, or co-infected with pairwise combinations or with all three viruses. After 48 h, soluble cytoplasmic and nuclear fractions were prepared and analyzed by immunoblotting. UL56 and UL89 were detected using polyclonal rabbit antisera and UL51 was detected using a FLAG epitope-specific mouse monoclonal antibody. To ensure efficient separation of nuclear from cytoplasmic material, blots were also probed with antibodies to the exclusively cytoplasmic protein (tubulin) or to the exclusively nuclear protein (histone H4).

**Figure 1:**
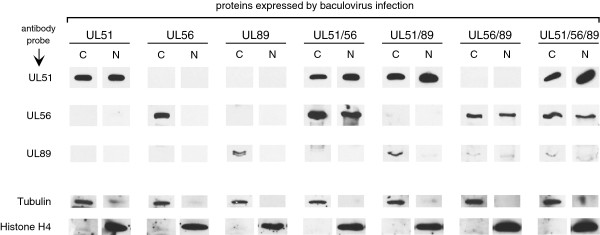
**Subcellular localization of terminase subunits expressed using recombinant baculoviruses.** Sf9 insect cells were infected at MOI = 3 with recombinant baculoviruses expressing UL56, UL89, or FLAG-UL51, as indicated. Soluble cytoplasmic (C) and nuclear (N) extracts were prepared 48 h post infection and analyzed by immunoblot using antibodies specific to each protein as indicated. Cytoplasmic and nuclear fractions were separated on different gels; for ease of comparison lanes from each are displayed side-by-side. In some blots anti-UL89 antibody cross-reacted with a cellular protein that migrated more slowly than UL89 (*e*.*g*., in the UL51/UL56 cytoplasmic extract).

When expressed alone, UL51 (17 kDa) was equally distributed between the cytoplasm and the nucleus, whereas UL56 (96 kDa) and UL89 (77 kDa) were found exclusively in the cytoplasm (Figure 1). Distribution of UL51 was not significantly affected by co-expression with other proteins; however, dual expression of UL56 with either UL51 or UL89 resulted in redistribution of about half of the UL56 to the nucleus. Similarly, dual expression of UL89 with UL56 resulted in redistribution of about half of the UL89 to the nucleus. When all three proteins were expressed, each protein appeared to be equally distributed between cytoplasmic and nuclear fractions (Figure 1).

**Figure 2:**
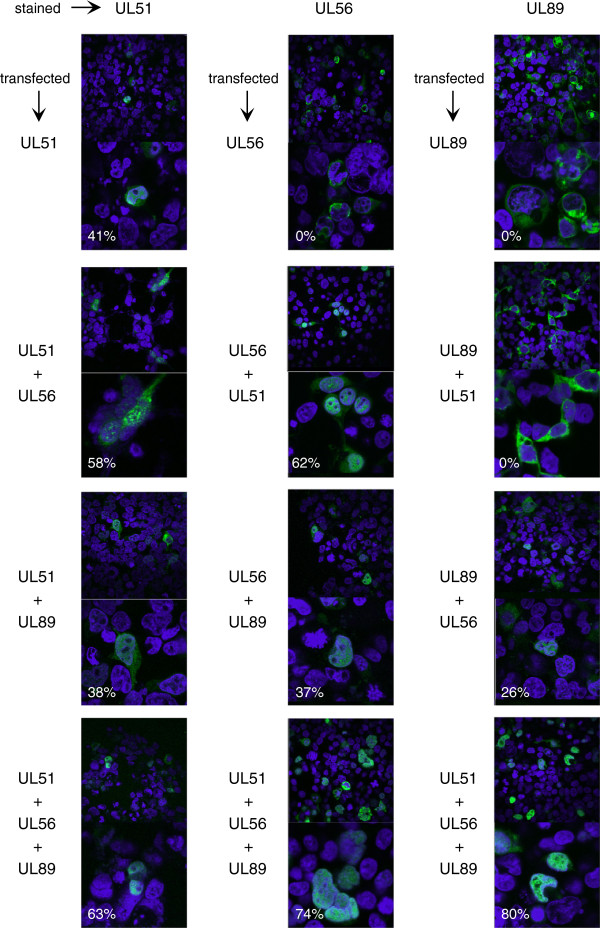
**Subcellular localization of terminase subunits expressed by plasmid transfection.** HEK-293 T cells were transfected with expression vectors encoding each terminase subunit fused to N-terminal FLAG (UL51), V5 (UL56), or MYC (UL89) epitope tags. 72 h post transfection the cells were fixed and stained with monoclonal antibodies specific to each epitope tag. The anti-V5 antibody was directly conjugated to FITC, whereas unconjugated primary antibodies for FLAG and MYC were detected using a FITC-conjugated anti-mouse IgG secondary antibody. Cells were counter stained with DAPI to detect nuclei. Paired images are representative micrographs taken at 0.7x (top) or 2x (bottom) zoom. In each column, cells were stained to detect the protein indicated above the column, while the expression vectors that were transfected are indicated to the left of each image pair. The percentages of cells with nuclear staining (in white) were determined by counting >50 fluorescent cells per group.

To determine if similar localization patterns occur in human cells, mammalian plasmid expression vectors were engineered to express UL51, UL56, or UL89 fused to N-terminal FLAG, V5, or MYC epitope tags, respectively. HEK-293 T cells were transfected with plasmid vectors individually or in combinations. After 48 h the cells were permeabilized and the subcellular localization of each protein was determined by confocal microscopy using fluorescent-tagged epitope-specific monoclonal antibodies (Figure 2). UL51 was often found in both the cytoplasm and the nucleus, with 41% of cells exhibiting nuclear staining. This proportion was not significantly altered by the presence of the other proteins. UL56 was exclusively cytoplasmic when expressed alone, but the percentage of cells with nuclear staining increased to 62% in the presence of UL51, 37% in the presence of UL89, and 74% when all three proteins were present. UL89 was also exclusively cytoplasmic when expressed alone, but unlike UL56, its localization was not affected by co-expression of UL51. However, the percentage of cells with UL89 nuclear staining increased to 26% in the presence of UL56 and to 80% when all three proteins were present (Figure 2).

## Discussion

The results from these experiments suggest that when expressed individually, both UL56 and UL89 are strictly cytoplasmic but co-expression promotes nuclear localization of both proteins. This could be a result of each protein harboring a partial nuclear localization signal (NLS) that is functional only when the two proteins interact, or due to protein interactions that alter conformation of at least one component to expose an otherwise hidden NLS. In either case, the data suggest that UL56 and UL89 interact. That UL56 can localize to the nucleus when co-expressed with UL51 also suggests that UL56 and UL51 interact in the cytoplasm to form a complex that can be transported to the nucleus. Again, interaction with UL51 could promote conformational changes that expose an NLS within UL56, or translocation of the complex could be mediated by an NLS in UL51. It is uncertain whether UL51 contains an NLS as its small size (~17 kDa) should allow nuclear entry independent of the nuclear pore complex.

While these results indicate that the two-subunit UL51/56 or UL56/89 complexes can form in the cytoplasm and subsequently localize to the nucleus, it cannot be ascertained from our studies whether all three proteins interact to form three-subunit UL51/56/89 complexes. Moreover, while we observed no evidence for UL51-UL89 interaction, it is possible that interactions were missed due to epitope tags on plasmid-expressed proteins or baculovirus-expressed UL51. A paper published following submission of this report confirms an essential role for UL51 in DNA packaging and demonstrates that each protein co-precipitates the other two [4]. Even so, co-precipitation or nuclear colocalization could be due to the ability of UL56 to interact independently with UL51 and UL89. However, if this were the case, the presence of UL51 should not increase the nuclear localization of UL89 in cells expressing all three proteins, but rather, UL51 might impair the nuclear localization of UL89 by competing for UL56 binding. In fact, in our studies nuclear localization of UL89, UL56, and UL51 all increased significantly when all three proteins were co-expressed in transfected 293 T cells (Figure 2).

**Figure 3:**
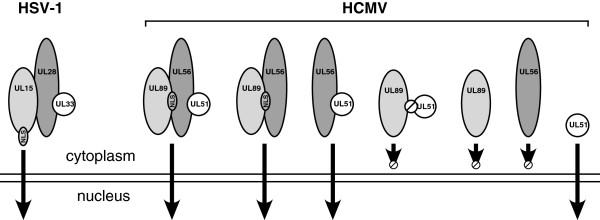
**Summary of terminase subunit interactions for HSV**-**1 and HCMV.** HCMV terminase subunit complexes that result in nuclear transport are illustrated. NLS, nuclear localization signal; Ø, no interaction, no nuclear translocation.

In agreement with our results, Borst et al. demonstrated that nuclear localization of UL89 and UL56 was severely diminished, though not apparently eliminated, in HCMV-infected cells when UL51 was knocked down [4]. Surprisingly, these authors were unable to detect cytoplasmic localization of UL89 and/or UL56 by immunofluorescence when UL51 protein was severely diminished. However, since knock down of UL51 did not impact immunoblot levels of UL89 or UL56, it is likely that immunofluorescence was not sensitive enough to detect these proteins when they were dispersed in the cytoplasm. Taken together, these data suggest that three-subunit complexes likely form in the cytoplasm and are more efficiently imported to the nucleus when all are present, because of cooperativity between the NLS formed between UL56 and UL89 and perhaps between UL56 and UL51, or because of conformational changes induced by interactions that expose a nuclear localization signal. The proposed interactions that effect transport of the terminase holoenzyme and its putative subunits are summarized in Figure 3.

Interactions between terminase subunits were first suggested by observations that the UL28 proteins of pseudorabies virus or HSV-1 are translocated to the nucleus when co-expressed with the UL15 protein of HSV-1 and that they co-purify as a complex in infected cells [5-7]. Subsequent studies implicated UL33 as an additional subunit of terminase complexes [8] and demonstrated direct UL28-UL15 and UL28-UL33 interactions and a UL28-dependent indirect interaction between UL15 and UL33 [9-13]. Our studies indicate functional interactions between HCMV orthologs UL56 and UL89 and between UL56 and UL51. Moreover, data reported by Borst et al. after our findings were submitted for publication indicate that UL51, UL89, and UL56 co-precipitate from HCMV-infected cell lysates, further suggesting that all three proteins exist in the same complex [4]. Interactions between terminase subunit orthologs of varicella zoster virus, Kaposi’s sarcoma-associated herpesvirus, and murine cytomegalovirus (MCMV) have also been reported [14-17].

The studies presented here are the first to characterize the intracellular trafficking of UL89 outside the context of infected cells and they directly implicate UL51 as a subunit of the HCMV terminase by virtue of its interaction with UL56. Borst et al. [4] also implicate UL51 as an essential member of the HCMV terminase complex since, despite normal levels of DNA replication, DNA cleavage and genome packaging into virions fail to occur when UL51 is knocked down in HCMV-infected cells. Because we were able to track localization of each subunit when expressed alone or with each of the other proteins, we were able to demonstrate an interdependence between UL56 and UL89 for nuclear transport even in the absence of UL51. This is consistent with prior reports of UL56-UL89 interaction and their co-localization to nuclear viral replication centers in HCMV-infected cells [2,3,18,19]. However, contrary to our findings, Giesen et al. detected UL56 in the nuclei of transfected COS7 cells in the absence of other HCMV proteins and mapped a putative NLS [18]. This may suggest that UL56 localization is cell type-dependent as UL56 failed to localize to the nuclei of insect cells or human HEK-293 T cells in our studies. It should be pointed out that neither our studies nor those of Borst et al. [4] have demonstrated direct protein-protein interactions between subunits despite the fact that they co-localize or co-precipitate. However, even if such interactions are indirect, our studies indicate that they do not involve an additional viral protein, although a cellular protein present in both insect and human cells could be required. Work is in progress to rule out such a possibility.

Thus, despite strong sequence conservation between orthologous terminase subunits, it appears that HCMV and HSV-1 have evolved subtle differences in nuclear import of terminase complexes. For HSV-1 import of terminase to the nucleus relies on an overt NLS in UL15, whereas in HCMV the orthologous subunit (UL89) clearly lacks nuclear transport capacity. Terminase instead appears to rely on nuclear import signals formed cooperatively between the three subunits. Nevertheless a common theme is emerging that terminase must assemble in the cytoplasm before it can move to the nucleus. Presumably this restriction serves an important purpose, perhaps preventing uncomplexed or incorrectly complexed subunits from exerting detrimental effects on the cleavage/packaging process.

The specific function of HCMV UL51 or its orthologs, remains unknown. Given that HSV-1 UL33 enhances the UL15-UL28 interaction [9] and that HCMV UL51 (or all three of the putative terminase subunits) is required for efficient nuclear localization of these subunits, a role for UL33 or UL51 orthologs in promoting or stabilizing optimal terminase conformation can be envisioned. Moreover, the report that the varicella zoster virus UL33 ortholog exhibits a plethora of interacting partners [16] has fueled speculation that UL33-family proteins may serve a more general role in stabilizing protein complexes [14]. Recent interactome analyses of the UL33 orthologs of MCMV and varicella zoster virus revealed interactions with tegument proteins as well proteins involved in transport of newly formed viral capsids across the nuclear membrane [17,20]. Such findings, combined with evidence that UL51 and its MCMV ortholog are present in virions [21,22], suggest that UL33-family proteins may mediate or stabilize protein-protein interactions that link DNA encapsidation with post-packaging processes of tegumentation and nuclear egress [17,20].

## Conclusions

The results support inclusion of UL51 as an HCMV terminase subunit and suggest that nuclear import of HCMV terminase may involve nuclear import signals that form cooperatively upon subunit associations. In recent years DNA maturation has emerged as a target for development of novel antivirals. Several “maturational” inhibitors have been identified [23-28]. Letermovir (AIC246), a recent candidate with potent activity against HCMV, has entered phase IIb clinical trials [29-32]. Dissection of the mechanisms of action of these inhibitors and identification of additional candidates would be considerably advanced by a better understanding of the structure and functions of terminase subunits and the holoenzyme.

## Methods

### Plasmid and baculovirus expression vector construction

Oligonucleotide primers used for vector construction are listed in Table 1. The UL51 coding sequence was PCR amplified from HCMV strain AD169 DNA with primer pair MOL269/MOL270 and ligated into pCR-XL-TOPO (Invitrogen) to make plasmid pMA319. An XbaI/HindIII fragment of pMA319 containing UL51 sequences was then ligated into XbaI/HindIII-restricted pFastbac1 (Invitrogen) to make plasmid pMA320. FLAG-UL51 expression plasmid pMA323 was constructed by PCR amplification of pMA320 with primer pair UL51-FLAG-H3F/UL51-FLAG-ER1R (which introduce flanking HindIII and EcoRI sites) and ligation of the HindIII/EcoRI-digested PCR product into HindIII/EcoRI-digested pcDNA-2FLAGAB, a modification of pcDNA3 to express N-terminal FLAG fusions [33]. Baculovirus shuttle plasmid pMA326 was constructed by PCR amplification of pMA323 with primer pair UL51-FLAG-BamHI/UL51-FLAG-ER1R (which introduce flanking BamHI and EcoRI sites) and ligation of the BamHI/EcoRI-digested PCR product into BamHI/EcoRI-digested pFastbac1.

**Table 1:** Oligonucleotides used for plasmid construction

***Plasmid***	***contains***	***Oligonucleotide***	***Sequence*** (***5***'-***3***')*
pMA319	UL51	MOL269	ATGTCCTGGGCTAAGCAGCG
		MOL270	TTATTTACCCGGCGCCGACT
pMA48	UL56 (5’)	MOL60	CCATGGAGATGAATTTGTTAC
		MOL64	CTCCTCGAGAATATGCTTGAT
pMA49	UL56 (3’)	MOL59	ACAGGTTAACGCAGACTACCA
		MOL65	ATATTCTCGAGGAGATTCGAC
pUL89	UL89 cDNA	MOL53	CTGCTGCGTGCCTAGCTGAC
		MOL54	CATCATGTTGCGCGGAGACT
pMA323	FLAG-UL51	UL51-FLAG-H3F	GACCTAAGCTTTCCTGGGCTAAGCAGCGGGTG
		UL51-FLAG-ER1R	TACGATGAATTCTTATTTACCCGGCGCCGACTC
pMA326	FLAG-UL51	UL51-FLAG-BamHI	CGGGATCCATGGACTACAAGGACGACGA
		UL51-FLAG-ER1R	TACGATGAATTCTTATTTACCCGGCGCCGACTC
pMA325	UL56	UL56-V5-F	GAGATGAATTTGTTACAGAAACTA
		UL56-V5-R	TTAACGCAGACTACCAGGCAC
pMA324	MYC-UL89	linkA	GATCCACCATGGAACAAAAACTCATCTCAGAAGAGGATCTGG
		linkB	AATTCCAGATCCTCTTCTGAGATGAGTTTTTGTTCCATGGTG
		UL89-NMyc-ER1F	CATGATGAATTCTTGCGCGGAGACTCGGCC
		UL89-NMyc-XB1R	TACATGTCTAGACTAGCTGACCCTGAAACG

*restriction sites used for cloning are underlined.

The UL56 coding sequence was assembled by PCR amplification of HCMV strain AD169 DNA using primer pairs MOL60/MOL64 and MOL59/MOL65 and cloning of the products into pGEM-T vector (Promega) to generate plasmids pMA48 and pMA49, respectively. An NcoI/XhoI fragment of pMA48 was then ligated into NcoI/XhoI-restricted pFastbac-HTa (Invitrogen) to make plasmid pMA50 and an NcoI fragment from pMA48 was inserted into NcoI-digested pMA50 make plasmid pMA51. An XhoI/SalI fragment from pMA49 was then inserted into XhoI-digested pMA51 to make baculovirus shuttle plasmid pMA52. V5-UL56 expression plasmid pMA334 was constructed by PCR amplification of pMA52 with primer pair UL56-V5-F/UL56-V5-R, cloning the product into pCR8/GW/TOPO (Invitrogen) to make plasmid pMA325, and LR clonase transfer of the insert to pcDNA3.1/nV5-DEST (Invitrogen).

The cDNA of UL89 was prepared by RT-PCR of RNA isolated from MRC-5 cells 72 hours post infection with HCMV strain AD169 using ULTRASPEC RNA (BiotecX). The RT-PCR reaction used SuperScriptTM II RNase H reverse transcriptase (Gibco BRL) and primer pair MOL53/MOL54. The product was cloned into pGEM-T vector to make plasmid pUL89. An SpeI/SphI fragment of pUL89 containing UL89 cDNA sequences was then ligated into SpeI/SphI-restricted pFastbac1 to make baculovirus shuttle plasmid pMA38. MYC-UL89 expression plasmid pMA324 was constructed by ligation of annealed oligonucleotides linkA and linkB into EcoRI/BamHI-restricted pcDNA4/TO (Invitrogen) to make pcDNA4/TO-c-myc followed by insertion of an EcoRI fragment containing HSV-1 *US11* to make pCDNA4/TO-myc-US11. *US11* sequences were then removed by EcoRI/XbaI digestion and replaced with *UL89* coding sequences by ligation to an EcoRI/XbaI-digested PCR product amplified from pMA38 using primer pair UL89-NMyc-ER1F/UL89-NMyc-XB1R (which introduce flanking EcoRI and XbaI sites). All expression vectors were verified by sequencing.

Baculovirus shuttle plasmids pMA326, pMA52, and pMA38 were used as directed in the BAC-to-BAC baculovirus system (Invitrogen, Grand Island, NY) to construct recombinant baculoviruses expressing FLAG-UL51, 6xhis-UL56, and UL89, respectively.

### Immunoblotting

Sf9 insect cells were infected with 3 pfu/cell of each baculovirus individually, or co-infected with pairwise combinations or with all three viruses. After 48 h, cytoplasm was separated from nuclei by dounce homogenization in hypotonic buffer followed by low-speed centrifugation as described [34]. Cytoplasmic supernatants were adjusted to 1 μg/ml aprotinin, leupeptin, and pepstain. Nuclei were suspended in 2 packed volumes of 20 mM Tris-HCl, pH8.2, 2 M NaCl, 2 mM EDTA, 2 mM 2-mercaptoethanol, 0.5 mM phenylmethylsulfonylfluoride and rocked gently at 4°C for 30 min. Nuclear fractions were clarified by centrifugation at 70,000 × *g* for 30 min. at 4°C. Soluble nuclear and cytoplasmic extracts were separated by SDS-PAGE and electrophoretically transferred to nitrocellulose membranes. Membranes were probed with rabbit antisera to UL56 (WAR8, raised against an *E*. *coli*-expressed GST fusion to UL56 residues 383-850, a gift from Tom Jones), UL89 (WAR21, raised against an *E*. *coli*-expressed GST fusion to UL89 residues 1-296, a gift from Tom Jones), or histone H4 (ab 10158, Abcam), or with mouse monoclonal antibodies to FLAG (F3165, Sigma) or tubulin (ab 6161, Abcam). Blots were developed using goat anti-mouse (Jackson Immunotherapeutics) or anti-rabbit (Thermo) IgG conjugated to horse radish peroxidase and the SuperSignal West Pico (GE Healthcare) luminescent substrate, followed by exposure to X-ray film.

### Transient expression and confocal microscopy

HEK-293 T cells were transfected with plasmid vectors individually or in combinations using Effectene (Qiagen). After 48 h the cells were permeabilized with cold methanol, blocked with phosphate buffered saline containing 1% BSA, and stained either with fluorescein isothiocyanate (FITC)-conjugated anti-V5 monoclonal antibody (Invitrogen), or unconjugated anti-MYC (Sigma) or M2 anti-FLAG monoclonal antibody (Sigma) followed by a FITC-conjugated anti-mouse IgG1 (Serotec) secondary antibody. Cells were counterstained with 1 μg/ml of 4', 6-diamidino-2phenylindole (DAPI). Images were collected using an LSM 510 Meta confocal laser scanning microscope with 63X oil immersion objective (numerical aperture 1.4), pinhole 0.7 μm, and excitation of 488 nm (FITC) or 405 nm (DAPI).

## Abbreviations

HSV-1: Herpes simplex virus type 1; HCMV: Human cytomegalovirus; NLS: Nuclear localization signal; MCMV: Murine cytomegalovirus; FITC: Fluorescein isothiocyanate; DAPI: 4', 6-diamidino-2phenylindole (DAPI).

## Competing interests

The authors declare that they have no competing interests.

## Authors’ contributions

JBW constructed the plasmid and baculovirus expression vectors and conducted the transient expression/confocal microscopy experiments. YZ conducted the baculovirus expression/immunoblot experiments. MM and DP conceived the experiments, interpreted the results, and prepared an initial draft of the manuscript. All authors were involved in revising the manuscript and have read and approved the final manuscript.
